# Annual Hospital Volume of High Dose Interleukin-2 and Inpatient Mortality in Melanoma and Renal Cell Carcinoma Patients

**DOI:** 10.1371/journal.pone.0147153

**Published:** 2016-01-22

**Authors:** Kathan Mehta, Leonard Appleman, Hong Wang, Ahmad A. Tarhini, Rahul A. Parikh

**Affiliations:** 1 University of Pittsburgh Medical Center, Pittsburgh, PA, United States of America; 2 University of Pittsburgh Cancer Institute, Pittsburgh, PA, United States of America; The Ohio State University, UNITED STATES

## Abstract

**Background:**

Immunotherapy using high dose interleukin-2 (HD IL2) in patients with renal cell carcinoma (RCC) and melanoma is associated with severe toxicities. The association between annual hospital volume of HD IL2 and inpatient mortality is not well studied. In this study we aim to quantify the impact of annual hospital volume of HD IL2 on inpatient mortality using National Inpatient Sample (NIS) data.

**Methods:**

We did a cross-sectional study using NIS, one of the largest inpatient datasets in United States, from 2003 to 2011. Patients with melanoma and RCC receiving HD IL2 were identified by ICD9 procedure code 00.15. The primary outcome was inpatient mortality. Using Joinpoint regression, which detects change in trend of inpatient mortality with change in annual volume, the hospitals were classified in three volume categories (low: 1–40, medium: 41–120, high: >120). Multivariate logistic regression was used to identify predictors of inpatient mortality controlling for confounders.

**Results:**

From 2003 to 2011, 29,532 patients with RCC or melanoma who received HD IL2 were identified, and 124 died during the hospitalization (0.4%). The hospitals with low, medium and high annual volume had significant difference in inpatient mortality (0.83%, 0.29% and 0.13% respectively, p = 0.0003). On multivariate analysis, low volume hospitals were associated with significantly higher odds of inpatient mortality (OR 6.1, 95% CI 1.6–23.2, p = 0.003) as compared to high volume hospitals. Additionally, the hospitals with annual volume of 1–20 had even higher rates (1.31% vs. 0.13%, p<0.0001) and multivariate odds (OR 8.9, 95% CI 2.4–33.2, p = 0.0006) of inpatient mortality as compared to high volume hospitals.

**Conclusions:**

Lower annual hospital volume of HD IL2 is associated with worse outcomes. Annual hospital volume of 1–40 and 1–20 treatments per year is associated with 6 and 9 times higher odds of inpatient mortality respectively as compared to high volume hospitals. Our findings provide preliminary evidence for a volume-outcome relationship for RCC and melanoma patients undergoing HD IL2 treatment. They support future volume-outcome analyses in relation to other anti-cancer therapies that require special training and expertise.

## Introduction

HD IL2 is used in treatment of selected patients with metastatic renal cell carcinoma (RCC) and metastatic melanoma due to its ability of inducing complete and durable responses in these patients.[1] Initial studies had shown that HD IL2 leads to partial response in 10% of patients and complete response in 6% of patients with metastatic melanoma and an overall response rate of 14% in renal cell carcinoma.[2,3] In a large prospective, multicenter study with one hundred and twenty seven evaluable subjects (Select), HD IL2 led to partial responses in 23% and complete response in 3% of patients with metastatic RCC.[4] These responses were seen in both good and poor risk subjects and positively associated with tumor PD-L1 expression.[4] HD IL2 use is associated with hypotension, cardiac arrhythmias, myocardial infarction, capillary-leak syndrome, reduced organ perfusion and rarely death. Death related to sepsis during HD IL2 was seen in approximately 2% of melanoma patients in a retrospective analysis.[5] These significant HD IL2 associated toxicities have limited its use to specialized centers. Despite several new options in the treatment of metastatic melanoma and RCC, HD IL2 continues to be widely used as salvage therapy. There are several ongoing clinical trials testing HD IL2 in combination with newer agents in patients with metastatic melanoma and RCC. The 2014 expert consensus on HD IL2 states that "Treating a minimum number of patients per year is important, as quality depends upon familiarity and repetition".[6] A clear association between annual volume of HD IL2 and clinical outcomes has not been studied. We hypothesized that higher annual HD IL2 volume is associated with lower inpatient death rates during HD IL2 administration.

## Material and Methods

We used the National Inpatient Sample (NIS), one of the largest publicly available inpatient dataset in United States (U.S.) for our analysis. NIS is published by Healthcare Cost and Utilization Project (HCUP), Agency for Healthcare Research and Quality.[7] NIS represents a 20% stratified random sample of discharges from all hospitals, excluding rehabilitation and long-term acute care hospitals. NIS is drawn from all States participating in HCUP, representing more than 95 percent of the U.S. population and uses National Health Survey Strata to weigh each participating hospital. Discharge weights are provided for each entry and are used to project to a nationally representative population. The details of NIS methodology have been previously published by the HCUP.[8]

We queried NIS database between 2003 and 2011 for patients with melanoma and RCC by using international classification of diseases 9^th^ edition–clinical modification (ICD9-CM) diagnostic codes published by HCUP.[9] From this sample, patients receiving IL2 were identified by ICD9-CM procedure code 00.15. Annual hospital volume was calculated by the unique hospital number available in the dataset. Using join-point regression software, which detects change in trend of inpatient mortality with change in annual hospital volume, the hospitals were classified in three volume categories (low, medium and high). Join-point regression uses the grid search method to detect points at which significant changes in the direction and magnitude of trend of dependent variable (mortality) with reference to the independent variable (hospital volume) occur, under the assumption of constant variance and uncorrelated errors.[10] The severity of co-morbid conditions was quantified by calculating the Deyo’s modification of Charlson co-morbidity index (CCI).[11,12] The index ranges from 0–33, with higher scores indicating severe co-morbid conditions. Since all patients in our sample had cancer, we excluded cancer from the calculation of index.

Chi-square test was used to test significance of difference in categorical variables. One-way ANOVA was used to test significance of difference in continuous variables. Multivariate logistic regression was used to identify predictors of inpatient mortality controlling for possible confounders (Age, sex, co-morbid conditions, type of malignancy (RCC vs. melanoma), calendar year, urban location of the hospital and teaching status of the hospital). Due to large amount of missing data, race was not included in the regression model. Using SAS 9.2 (SAS Institute Inc., Cary, NC, USA), SURVEY procedures were used to adjust for stratified design of NIS.

## Results

**Fig 1:**
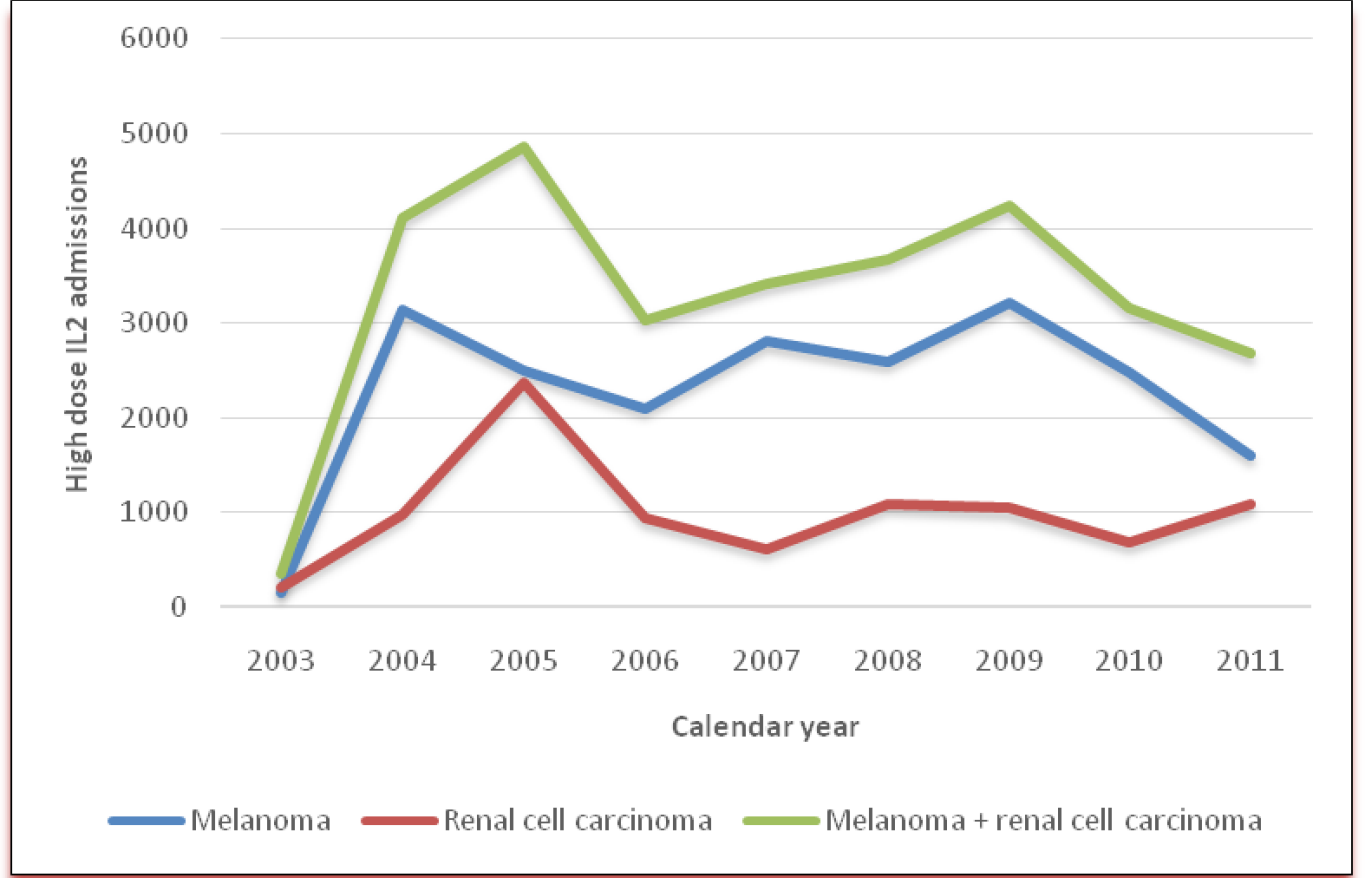
Utilization of high dose interleukin-2 (HD IL2) in United States from 2003 to 2011.

From 2003 to 2011, the NIS contained 29,532 patients (weighted number) with RCC (weighted N = 8,969) or melanoma (weighted N = 20,563) who received HD IL2. The annual number of HD IL2 treatments captured by NIS is described in Fig 1. Utilization of HD IL2 for RCC and melanoma appears to be declining after 2005 and 2009 respectively (Fig 1). The annual hospital volume for HD IL2 ranged from 1 treatment per year to 286 per year with the median of 71 per year. The Joinpoint regression classified the hospitals in 3 volume categories (low: 1–40, medium: 41–120, high: >120) based on change in trend of mortality. There was no difference in age, sex, and race of the patients in different hospital volume categories (Table 1). The high volume hospitals had lesser proportion of RCC patients (16.9% vs. 36.2% vs. 37.9%, p = 0.01) as compared to medium and low volume hospitals. The patients admitted to low volume hospitals had higher burden of co-morbid conditions (CCI of 0.3 vs. 0.1 vs. 0.1, p = 0.0004) as compared to medium and high volume hospitals. High volume hospitals were more likely to be in urban location as compared to medium and low volume hospitals (100% vs. 85.9% vs. 96.1%, p<0.001). There was no difference in teaching status of the hospitals among three volume categories.

**Table 1:** Baseline characteristics of melanoma and renal cell carcinoma patients undergoing high dose interleukin-2 treatment in United States from 2003 to 2011.

Characteristics	Annual hospital volume				
	0–40	41–120	> 120	P value	
Weighted N	9865	9876	9791		
Age (Mean ± SE)	52.3 ± 0.6	52.2 ± 0.8	50.4 ± 0.8		0.1
Female (%)	31.2	33.9	35.1	0.3	
Race (%)				0.2	
→White	73.5	58.4	89.6		
→African American	1.2	0.7	0.6		
→Hispanic	3.2	4.5	3.4		
→Other	3.7	2.0	2.0		
→Missing	18.4	34.4	4.3		
CCI ^a^ (Mean ± SE)	0.3 ± 0.03	0.1 ± 0.02	0.1 ± 0.02	0.0004	
Underlying malignancy				0.01	
→Renal cell carcinoma	37.9	36.2	16.9		
Primary payer (%)				0.009	
→Medicare / Medicaid	22.5	16.4	12.8		
→Private including HMO	71.0	81.1	80.1		
→Self-pay/no charge/other	6.4	2.5	7.1		
Hospital location (%)				<0.001	
→Urban	96.1	85.9	100		
Hospital teaching status (%)				0.8	
→Teaching hospital	83.6	88.4	84.8		
LOS (days) (Mean ± SE)	5.5 ± 0.1	4.7 ± 0.1	4.4 ± 0.6	<0.0001	
Total Charges ^b^ ($) (Mean ± SE)	93,376 ± 5,399	73,332 ± 7,750	61,813 ± 5,776	0.0002	

a. Abbreviations: CCI—Charlson co-morbidity index; HMO - health maintenance organization; LOS–length of stay.

b. Total charges for each year were adjusted for inflation by using consumer price index published by bureau of labor statistics.

**Fig 2:**
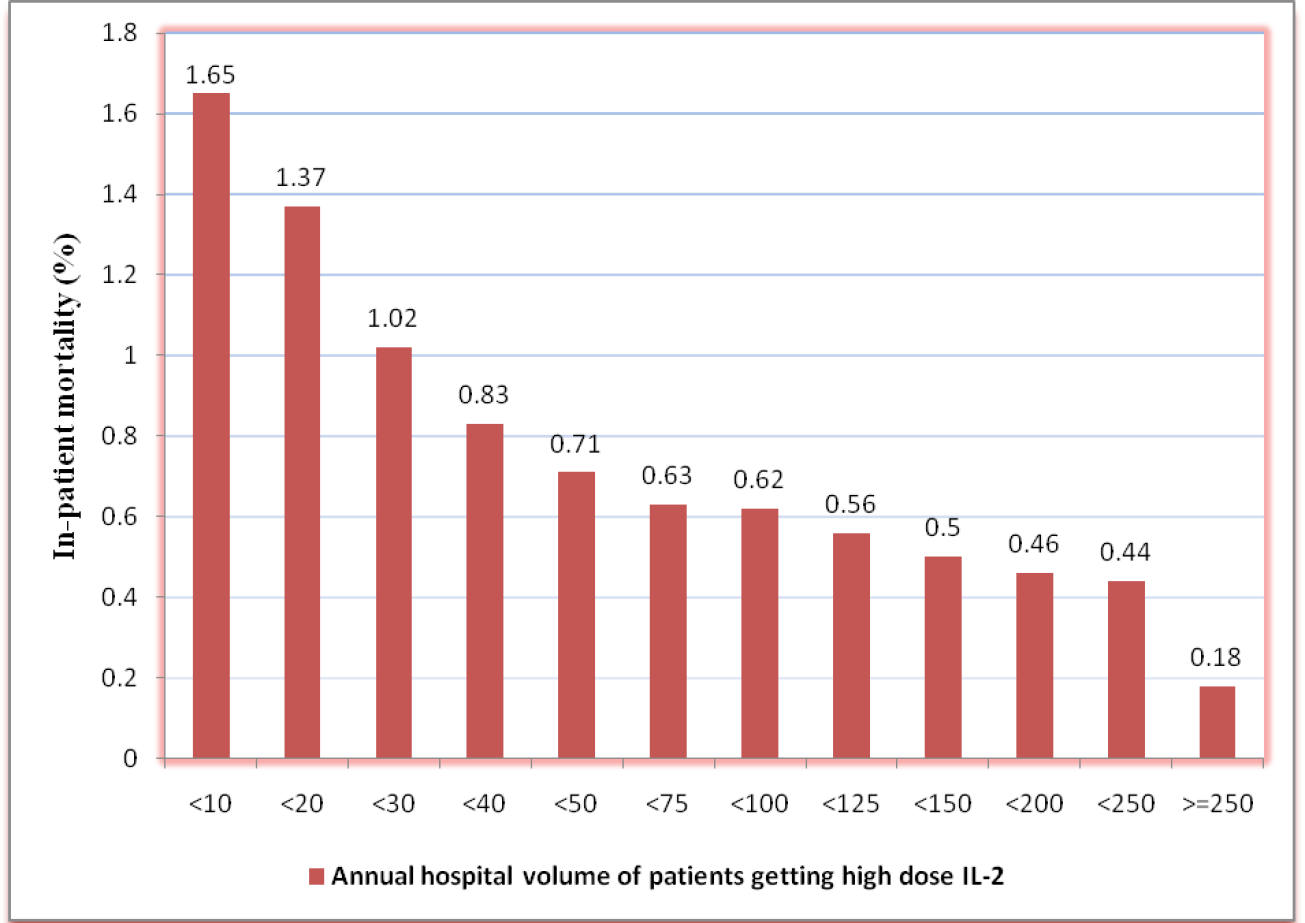
Impact of annual hospital volume of high dose interleukin– 2 admissions on in-hospital mortality. Annual hospital volume of high dose interleukin– 2 admissions is plotted as cumulative frequency on X–Axis.

Among the 29,532 patients receiving HD IL2, 124 died during the hospitalization (0.4%). The higher hospital volume cut-offs showed consistent reduction in inpatient mortality (Fig 2). The hospitals with low, medium and high annual volume had significant difference in inpatient mortality (0.83%, 0.29% and 0.13% respectively, p = 0.0003). On multivariate analysis, the low volume hospitals were associated with higher odds of inpatient mortality (OR 6.1, 95% CI 1.6–23.2, p = 0.003) as compared to high volume hospitals. Additionally, the hospitals with annual volume of 1–20 had even higher rates (1.31% vs. 0.13%, p<0.0001) and multivariate odds (OR 8.9, 95% CI 2.4–33.2, p = 0.0006) of inpatient mortality as compared to high volume hospitals. Age, sex, co-morbid conditions, type of malignancy (RCC vs. melanoma), calendar year, urban location of the hospital and teaching status of the hospital were not associated with increased inpatient mortality (Table 2). Additionally, the patients admitted to low volume hospitals had higher length of stay (5.5 days vs. 4.7 days vs. 4.4 days, p<0.0001) and total charges ($93,376 vs. $73,332 vs. $61,813, p = 0.0002) as compared to medium and high volume hospitals.

**Table 2:** Multivariate predictors of in-hospital mortality in melanoma and renal cell carcinoma patients receiving high dose interleukin-2 (weighted N = 29,184).

Variable	Odds Ratio (95% CI)	P value
Age	1.01 (0.98–1.04)	0.4
Female gender	0.46 (0.17–1.27)	0.1
Deyo modification of CCI ^a^	1.39 (0.99–1.94)	0.06
Renal cell carcinoma	0.81 (0.36–1.82)	0.6
Calendar year	0.92 (0.78–1.1)	0.4
Urban location of hospital	0.65 (0.14–2.97)	0.6
Teaching hospital	0.58 (0.22–1.51)	0.3
Hospital volume category		
→High (> 120 per year)	Referent	
→Medium (41–120 per year)	2.36 (0.52–10.7)	0.9
→Low (1–40 per year)	6.12 (1.61–23.17)	0.003

a. Abbreviations: CCI–Charlson co-morbidity index

## Discussion

Since the Food and Drug Administration’s (FDA) approval of HD IL2 for RCC in 1992[13] and for melanoma in 1998,[14] it has been widely used in treatment of both malignancies. Since approval of Sorafenib (2005)[15] and Sunitinib (2006)[16] by FDA, the utilization of HD IL2 has reduced for RCC patients (Fig 1). Ipilimumab, which inhibits cytotoxic T-lymphocyte-associated antigen 4 was shown to provide survival advantage, with or without a gp100 peptide vaccine, as compared with gp100 alone, in patients with previously treated metastatic melanoma in 2010;[17]. This lead to FDA approval for ipilimumab in 2011.[18] FDA also approved Vemurafenib in 2011 and dabrafenib for melanoma patients with the BRAF V600E mutation.[19–21] Currently, the standard of care for patients with metastatic melanoma with the BRAF V600E mutation is a combination of BRAF and MEK inhibitors.[22,23] After introduction of these new therapies the utilization of HD IL2 has reduced for melanoma patients. While most of initial experience with HD IL2 was at National Cancer Institute, gradually more and more hospitals have started treating patients with HD IL2 therapy. Currently, there are more than 60 centers in the U.S. offering IL2 therapy.[6] The management of HD IL2 therapy and treatment related adverse events vary considerably among centers. It has been argued that the centers with more experience in HD IL2 may perform better patient selection and manage toxicities with more diligence leading to lower treatment related mortality.[6] Data presented in this study clearly shows that higher hospital volume is associated with lower mortality. Moreover, the patients selected for HD IL2 treatment at lower volume centers may have higher co-morbid conditions, which may have contributed to higher mortality. The higher length of stay and higher total charges at low volume hospital could be attributed to higher complication rates. Thus, the results of this study lend evidence to support the hypothesis of volume-outcome relationship of HD IL2 treatment.

The volume-outcome relationship is well studied in surgical procedures like pancreatectomy and esophagectomy.[24] Prior studies have shown similar volume outcome relationship in allogeneic bone marrow transplant.[25,26] However, the data on volume-outcome relationship of other medical treatments for cancers is very limited. As a result, the Institute of Medicine’s workshop on volume-outcome relationship identified cancer treatment and non-surgical interventions as a new area of research.[27] To our knowledge, this study is the first study to report volume-outcome relationship of HD IL2 therapy. Our study has important implications for RCC and melanoma patients who are considering HD IL2 treatment and physicians who are referring their patients for HD IL2 therapy at specialty centers. With decrease in overall utilization of HD IL2 and increase in number of centers offering HD IL2 therapy, the annual hospital volume of HD IL2 treatments may decline in near future. This may pose a unique challenge to our healthcare system, to provide safe and high quality care to our patients who choose to undergo HD IL2 treatments.

Our study has several limitations. The NIS does not contain the number of the HD IL2 doses received or number of doses missed by the patient. While the NIS contains a variable for the attending physician, we could not study the impact of annual physician volume of HD IL2 treatment on outcomes due to large amount of missing data. Recently, there is a shift to offer a number of therapies including HD IL2 as outpatient. Due to the nature of the NIS database, we are unable to capture this information and compare it to inpatient HD IL2 use. Due to lack of post-discharge follow up data, we could not study the overall survival. Finally, the NIS does not contain data on vital signs, laboratory values, radiological data, EKG or weight of the patient. Despite these limitations, our study represents the largest sample of patients, who have received HD IL2 for melanoma or RCC.

## Conclusions

Lower annual hospital volume of HD IL2 is associated with worse outcomes. Annual hospital volume of 1–40 and 1–20 treatments per year is associated with 6 and 9 times higher odds of inpatient mortality respectively as compared to high volume hospitals. Our findings provide preliminary evidence for a volume-outcome relationship for RCC and melanoma patients undergoing HD IL2 treatment. They support future volume-outcome analyses in relation to other anti-cancer therapies that require special training and expertise.

## Abbreviations

HD IL2: High dose interleukin-2
RCC: Renal cell carcinoma
NIS: National inpatient sample
U.S.: United States
HCUP: Healthcare cost and utilization project
ICD9-CM: International classification of diseases 9^th^ edition–clinical modification
CCI: Charlson co-morbidity index
FDA: Food and Drug Administration
